# In Vitro Study of Extracellular Vesicles Migration in Cartilage-Derived Osteoarthritis Samples Using Real-Time Quantitative Multimodal Nonlinear Optics Imaging

**DOI:** 10.3390/pharmaceutics12080734

**Published:** 2020-08-05

**Authors:** Leonardo Mortati, Laura de Girolamo, Carlotta Perucca Orfei, Marco Viganò, Marco Brayda-Bruno, Enrico Ragni, Alessandra Colombini

**Affiliations:** 1 INRIM-Istituto Nazionale di Ricerca Metrologica, 10135 Torino, Italy; l.mortati@inrim.it; 2 IRCCS Istituto Ortopedico Galeazzi, Laboratorio di Biotecnologie Applicate all’Ortopedia, via R. Galeazzi 4, 20161 Milano, Italy; laura.degirolamo@grupposandonato.it (L.d.G.); carlotta.perucca@grupposandonato.it (C.P.O.); marco.vigano@grupposandonato.it (M.V.); alessandra.colombini@grupposandonato.it (A.C.); 3 IRCCS Istituto Ortopedico Galeazzi, III Spine Surgery—Scoliosis Department, via R. Galeazzi 4, 20161 Milano, Italy; marco.brayda@spinecaregroup.it

**Keywords:** osteoarthritis, cartilage, mesenchymal stem cells, extracellular vesicles, coherent anti-stokes raman scattering, second harmonic generation, two-photon excitation fluorescence, time-lapse, microscopy

## Abstract

Mesenchymal stromal cells (MSCs)-derived extracellular vesicles (EVs) are promising therapeutic nano-carriers for the treatment of osteoarthritis (OA). The assessment of their uptake in tissues is mandatory but, to date, available technology does not allow to track and quantify incorporation in real-time. To fill this knowledge gap, the present study was intended to develop an innovative technology to determine kinetics of fluorescent MSC-EV uptake by means of time-lapse quantitative microscopy techniques. Adipose-derived mesenchymal stromal cells (ASCs)-EVs were fluorescently labeled and tracked during their uptake into chondrocytes micromasses or cartilage explants, both derived from OA patients. Immunofluorescence and time-lapse coherent anti-Stokes Raman scattering, second harmonic generation and two-photon excited fluorescence were used to follow and quantify incorporation. EVs penetration appeared quickly after few minutes and reached 30–40 μm depth after 5 h in both explants and micromasses. In explants, uptake was slightly faster, with EVs signal overlapping both extracellular matrix and chondrocytes, whereas in micromasses a more homogenous diffusion was observed. The finding of this study demonstrates that this innovative technology is a powerful tool to monitor EVs migration in tissues characterized by a complex extracellular network, and to obtain data resembling in vivo conditions.

## 1. Introduction

The immune/inflammatory-mediated joint degeneration in osteoarthritis (OA) can be counteracted by mesenchymal stromal cells (MSCs), mainly through the secretion of immunomodulatory and trophic paracrine factors, such as proteins, lipids and nucleic acids, both free and conveyed within extracellular vesicles (EVs), also shuttling miRNAs [1,2]. MSC-EVs share regenerative, anti-inflammatory and immunomodulatory biological functions with releasing cells [3,4], thus representing a promising cell-free tool for the treatment of OA, as observed for EVs released from human bone marrow, adipose, embryonic or synovium MSCs in vitro [5] and in vivo [6,7,8,9]. 

These encouraging experimental evidences paved the way towards the molecular dissection and future engineering of the EVs, to obtain both natural or empowered therapeutic vehicles suitable for the treatment of OA. In this regard, three fundamental aspects are mandatory to predict clinical relevant results in patients: (i) the definition of their content through high-capacity platforms and identification of therapeutic molecules, (ii) the quantification of the amount of these naturally occurring or exogenously loaded factors and (iii) the verification of specific uptake in the target tissues and cells with a defined kinetics. The first two issues have been largely stressed in the past years, especially for MSC-EVs [3]. The last one, although clearly appearing as a crucial parameter to be established for a successful treatment, has been largely underestimated. Few studies have characterized the process and kinetics of MSC-EV uptake, mainly in vitro and in two-dimensional (2D) cultures, and even a smaller number in the musculoskeletal field [10,11,12]. Traditional tissue analyses such as conventional histology and immunohistochemistry are valuable tools, albeit only allowing an endpoint evaluation at the price of major processing of the tissues, together with loss of the tridimensional structure. A more refined and in vivo resembling model based on non-destructive approaches that allows a real-time and quantitative analysis of tridimensional biological structure is needed to evaluate the EVs migration at both tissue and cellular level.

Innovative multiphoton microscopy techniques are an intriguing alternative that allows tridimensional imaging and a deeper penetration into thick constructs and tissue samples [13]. In this frame, a combination of coherent anti-Stokes Raman scattering (CARS) and second harmonic generation (SHG) with two-photon excited auto-fluorescence (TPEAF) microscopy was recently used for quantitative image analysis to assess the engineered cartilage formed by human skeletal cells and monitor the interaction between collagens and chondrocytes over time [14]. Raman spectroscopy combined with SHG techniques was also used to evaluate both the spatial biochemical distribution and collagen fiber orientation across the full thickness of bovine articular cartilage [15]. Moreover, three-dimensional (3D) quantitative image data on diseased and treated murine joints were obtained using confocal laser scanning microscopy (CLSM) and micro-computed tomography (μCT) in order to assess the therapeutically effect through the tissue morphology variation [16]. The dynamic of the skeletal morphogenesis and cartilage growth in an avian model has been studied in a time-lapse experiment using the two-photon laser scanning microscopy [17]. Multimodal nonlinear optical (NLO) microscope system integrating stimulated Raman scattering (SRS), SHG and two-photon excited fluorescence (TPEF) have been used to study the functions of kinesin-1 in cartilage development in fresh tibial cartilage from normal and mutant mice at different developmental stages using [18]. Based on these findings, NLO microscopy technology could also be exploited to assess fluorescently-labeled EVs migration in tridimensional human cartilaginous structures at cellular level in a time-lapse experiment.

As important as the imaging techniques, the choice of appropriate biological samples to be used for the correct assessment of EV uptake and kinetics is challenging. Micromass (or pellet culture) have been extensively applied as an in vitro model of cartilage formation [19,20]. Nevertheless, despite being the most commonly used and well-known model of in vitro chondrogenic differentiation, the pellet culture presents several limitations. The need of a high number of cells to prepare micromasses account for an extensive monolayer expansion of the scarce and low-proliferating chondrocytes isolated from native cartilage, leading inevitably to the loss of the physiological cell phenotype [21,22,23]. After 2D expansion, cells packed in micromass need a further induction toward the native phenotype through 28 days of maintenance under chondrogenic induction, with the limited nutrient supply of the core often leading to a progressive central necrosis of the structures [24]. Moreover, the huge number of cells in micromasses, usually 400,000–500,000 chondrocytes for approximately 1 mm^3^ mass, is not representative of the physiological scarce cellularity of cartilage, i.e., around 10,000 cells per 1 mm^3^ in cartilage layer covering the medial femoral condyle [25]. Additionally, the production of type II collagen is substantially lower in comparison with the native tissue [26,27]. To overcome these technical and biological limitations, ex vivo models used in OA research are represented by cartilage explant, better replicating the extracellular matrix environment of native articular cartilage [20,28]. 

The aim of the present study is the development of an experimental model to determine the kinetics of fluorescent MSC-EVs migration and distribution in micromasses and cartilage explants that are the most representative of the tissue physiology, examined by means of new real-time, quantitative imaging techniques.

## 2. Materials and Methods 

### 2.1. Cartilage and Adipose Tissue Collection, Cell Isolation and Expansion

The study protocol was conducted in accordance with the Declaration of Helsinki and approved by the local Institutional Review Board (San Raffaele Hospital Ethics Committee approval on date 8 March 2018, registered under number 6/int/2018) and tissue samples were collected after obtaining patient’s informed written consent. 

Articular cartilage slices of about 100–250 μm depth and 3–5 mm length were collected with a scalpel from the non-weight bearing areas of femoral heads/necks of two females donors and one male donor affected by OA (Kellgren Lawrence III–IV, 38–54 years old) undergoing total hip arthroplasty. All donors have been processed as follows. Part of the slices were individually maintained in culture medium and used for kinetic tests; the remaining cartilage was pooled and enzymatically digested to isolate chondrocytes (37 °C, 22 h with 0.15% *w/v* type II collagenase (Worthington Biochemical, Lakewood, NJ, USA)) [29]. Subcutaneous adipose tissue was harvested from liposuction of three female donors (46–62 years old). Adipose-derived MSCs (ASCs) were isolated by enzymatic digestion (37 °C, 30 min with 0.075% *w/v* type I collagenase (Worthington Biochemical, Lakewood, NJ, USA)) [30]. After digestion, cartilage explants and both cell types were cultured in high glucose (4.5 mg/mL) DMEM, supplemented with 10% FBS (Lonza), 0.29 mg/mL L-glutamine, 100 U/mL penicillin, 100 μg/mL streptomycin (complete medium). Chondrocyte cultures were further supplemented with 10 mM 4-(2-hydroxyethyl)piperazine-1-ethanesulfonic acid (HEPES) and 1 mM sodium pyruvate (all reagents from Thermo Fisher Scientific Waltham, MA, USA).

Cartilage explants were used within two days from collection while ASCs and chondrocytes were expanded for three passages at 37 °C, 5% CO_2_ and 95% humidity.

### 2.2. ASCs Characterization by Flow Cytometry

A CytoFlex (Beckman Coulter, Fullerton, CA, USA) flow cytometer, collecting a minimum of 30,000 events, was used to confirm ASCs phenotype [31]. The following antibodies were used: CD90-FITC (REA897), CD73-PE (REA804), CD34-FITC (AC136), CD45-PEVIO770 (REA747) and CD31-PERCPVIO700 (REA730) (Miltenyi, Bergisch Gladbach, Germany) and CD44-PERCP (44PP2) (Immunostep, Salamanca, Spain). Incubation was allowed for 30 min in the dark at 4 °C, as per the manufacturer’s instructions.

### 2.3. Extracellular Vesicles (EVs) Isolation and Staining 

ASCs at 90% confluence were washed twice with PBS before adding serum free medium (10 mL per T175 flask). Culture supernatants were collected after 48 h and cleaned from floating cells, debris and apoptotic bodies by differential centrifugation at 4 °C at 376× *g*—15′, 1000× *g*—15′, 2000× *g*—15′ and twice at 4000× *g*—15′. Equal volumes of conditioned media from the three ASCs were pooled and, when needed, stained with 10 μM CFSE at 37 °C for 1 h in the dark. EVs were collected by ultracentrifugation with a 70Ti rotor (Beckman Coulter, Fullerton, CA, USA) for 3 h at 110,000× *g* at 4 °C. Pellet was washed with ice-cold PBS and additionally centrifuged for 1h at 110,000× *g* at 4 °C. Pellet was suspended in DMEM at a ratio of 100 μL per initial 10 mL of pooled conditioned medium. EVs were used immediately or preserved at −80 °C until use.

### 2.4. EVs Quantification and Dimensional Evaluation by Nanoparticle Tracking Analysis (NTA)

EVs were 1:500 diluted in PBS and 1 mL analyzed with Nanosight LM10-HS system [32] (NanoSight Ltd., Amesbury, UK). Five recordings of 60 s were conducted for each EV sample and NTA dedicated software v3.2 used to provide both concentration measurements and high-resolution particle size distribution profiles.

### 2.5. EVs Characterization by Flow Cytometry

CFSE-stained EVs were 1:150 diluted in PBS and antibodies (Biolegend, San Diego, CA, USA) anti-CD9-APC (HI9A), CD63-APC (H5C6) and CD81-APC (5A6) for EV markers [33], and CD44-APC (BJ18), CD73-APC (AD2) and CD90-APC (5E10) as MSC markers [34] were added at 1:20 ratio; incubation was performed for 30 min at 4 °C in the dark. After a further 1:7 dilution with PBS, samples were analyzed with a CytoFlex flow cytometer comparing outcomes with those obtained running FITC (Fluorescein isothiocyanate)-fluorescent beads of 160, 200, 240 and 500 nm (Biocytex, Marseille, France). PBS supplemented with CFSE and unstained EVs were used as negative controls.

### 2.6. Transmission Electron Microscopy (TEM)

Five μL of PBS-suspended EVs were absorbed on Formvar carbon-coated grids for 10 min. Then, excess liquid was removed by a filter paper. Negative stain was performed with 2% uranyl acetate and after 10 min, excess was removed. The grid was dried at room temperature. EVs were examined with a TALOS L120C transmission electron microscope (Thermo Fisher Scientific, Waltham, MA, USA) at 120 kV.

### 2.7. Chondrocyte Micromass Cultures

After chondrocyte isolation from the donors, the pellets were obtained by centrifugation (2 min at 232× *g*) of 4 × 10^5^ chondrocytes, and afterwards maintained for 4 weeks in chondrogenic medium composed by serum-free complete medium, 1% non-essential amino acids, 25 mM HEPES, 1 mM sodium pyruvate, 1% ITS + 1, 10 ng/mL TGFβ1 (Peprotech, Rocky Hill, NY, USA), 100 nM dexamethasone and 50 μg/mL ascorbic acid 2-phosphate (all reagents unless otherwise specified from Sigma–Aldrich, St. Louis, MO, USA) [35].

### 2.8. EVs Incorporation Analysis by Standard Immunofluorescence

Micromasses of approximately 1–1.5 mm diameter were incubated without or with 15 × 10^9^ CFSE-labeled EVs in 600 μL complete chondrogenic medium (approximately 25 × 10^8^ EVs per μL) for 48 h and medium without or with the same number of EVs replaced and maintained for additional 48 h (in total 96 h of EVs culture). Afterwards, micromasses were fixed in formalin, embedded in paraffin and then cut into 4 μm sections for end-point immunofluorescence analysis. Fluorescent images were collected with an IX71 Olympus inverted microscope (Olympus, Tokyo, Japan).

### 2.9. EVs Incorporation Detection by Time-Lapse Microscopy

EV-micromass co-cultures were performed by using 25 × 10^8^ CFSE-labeled EVs per μL as aforementioned, and the same protocol was used for cartilage explants immediately after donor tissue processing. The samples were followed over a period of 5 h. The size of the imaged pellet portion was chosen with a planar XY section having a width of 312.12 µm (452 pixels) and a height of 311.43 µm (451 pixels) and the Z pitch was 2 µm for a total depth of 64 µm (32 Z slices). The pellet was measured each 30 min (0.5 h) for a total of 11 timeframes. The size of the imaged cartilage portion was chosen with a planar XY section having a width of 279.67 µm (405 pixels) and a height of 350.79 µm (508 pixels) and the Z pitch was about 10.5 µm for a total depth of 210.50 µm (20 Z slices). The cartilage was measured each 15 min (0.25 h) for a total of 22 timeframes. Three images per Z slice have been acquired, for a total of 96 and 60 images for pellet and cartilage, respectively.

The experimental setup of the multimodal microscope allowed to collect images with Coherent Anti-Stokes Raman Scattering (CARS) combined with Second Harmonic Generation (SHG) techniques, as previously described [36], together with Two-Photon Excitation Fluorescence (TPEF) images (Figure S1). 

The TPEF channel served to acquire the fluorescence of the EVs, the cellular lipidic structures were imaged by the CARS signal resonating at about 2848 cm^−1^ and eventually the collagen of the extracellular matrix by SHG signal. The master source for the multimodal microscope was a passively mode-locked 76 MHz Nd:YVO_4_ (Yttrium Vanadate crystal doped with Neodymium) laser, emitting 10 ps pulses centered at 1064 nm (Picotrain, High-Q, Hohenems, Austria). An Optical Parametric Oscillator (Levante Emerald OPO) (APE, Berlin, Germany) was synchronously pumped by the master source frequency doubled output at 532 nm (5 ps, 4 W) and acted as the tunable source (tuning range 700–1020 nm for the signal output and 1110–2200 nm for the idler output). CARS signal looked at the CH_2_ stretching mode (2848 cm^−1^), with CARS resonance generated tuning the OPO signal pulse at about 817 nm as the pump field and overlapping it in space and time with part of the 1064 nm pulse, which aced as the Stokes field. At the same time, TPEF and SHG images were collected using the same sources to generate their respective multi-photon processes from the sample. Overlapped signal and 1064 nm beam entered into the microscope scanning unit (FluoView FV300, Olympus, Tokyo, Japan) combined with an upright microscope (BX51WI, Olympus, Tokyo, Japan). Non-linear optical signals were detected scanning the samples point-by-point with high resolution and excitation efficiency. The xy pixel pitch was about 69 nm and the pixel dwell time was about 8 μs. The average power at the sample of Stokes plus pump pulses was set to about 40 mw, adjusting an achromatic half wave plate in combination with a calcite polarizer. A stepping motor moved synchronously the focusing objective during the acquisitions, enabling a Z depth scanning of the sample, and thus, its 3D reconstruction. A water immersion objective (LUMPLFLN 40XW NA = 0.8, W.D. = 3.3 mm, Olympus), fully compensating for both spherical and chromatic aberrations from the UV to the near infrared region, was used to focus the excitation beams on living samples. The water immersion objective was cleaned and sterilized with a solution 70% ethanol in water (*v/v*) before each imaging experiment to prevent cell culture damages. The forward CARS signal at about 663 nm and SHG signal at about 408 nm were collected through an objective (UPLSAPO 10x objective NA = NA = 0.4, Olympus) and split through a dichroic mirror that reflects in the optical range of 360–510 nm, and thus spectrally filtered in the range between 640–700 nm for the CARS signal and in the range between 395–415 nm for the SHG signal, getting rid of the unwanted spectral components. The CARS and SHG beams were then focused on two separate PMTs (R3896, Hamamatsu, Japan) with two plano-convex lenses with a focal length of 25 mm. On the other hand, the TPEF signal was optically filtered in the range 510–530 nm and acquired in a back-scattering geometry (epi-detection), using one of the two PMT detectors placed inside the microscope scanning head. The microscope was also equipped with a microscope stage incubator (Okolab, Italy) that maintained the temperature at 37 °C, the CO_2_ concentration at 5% and the humidity range between 50 and 95% through an Active Humidity Controller.

### 2.10. Multimodal Microscopy Data Analysis

3D images were processed using custom-made ImageJ plugins in order to extract the depth of EV penetration and their total occupied volume during the time-lapse. Both micromasses and explants experienced background autofluorescence in the green channel (Figure S2a), being lower for the micromass. To minimize the autofluorescence interference for the EV detection, the Z stack of the TPEF signal measured at time 0 was subtracted pixel by pixel on all the other time-lapse images. To extract the information related to the EV depth of penetration, the foreground (the EVs) was separated from the background by choosing a suitable threshold level (Figure S2b). The binary images were then filtered to reduce the salt and pepper noise effect, by eliminating the objects with a surface smaller than a defined area considering the actual limit of data significance (Figure S2c). The chosen smallest allowed particle size was 100 pixels for both experiments.

Then, the number of binary Z slices distanced by a defined Z pitch was computed where the EVs pixels of the XY planes were detected, creating a new topographic image for each time-lapse step, representing the computed values of the depth as an intensity level in grey scale. From these images the localization of thickness in the sample during the time-lapse were extracted, together with the average penetration depths, the average occupied areas and thus the occupied volumes of the EVs over time, giving more tools to insight the understanding of the EV uptake dynamics. The average penetration depths and the average occupied areas were computed in each time-lapse step averaging only those voxels with a depth larger than zero. The occupied volume was computed summing up all the voxels volumes with a depth larger than zero. The extracted parameters were based on the idea to refer the EVs presence only where there was an actual presence of biological species in the image neglecting the background shape influence. By analyzing the dynamic of these parameters, it was possible to extract the time constants of the uptake dynamic exponential transients of the penetration depth, the occupied area and the occupied volume. Moreover, for the cartilage sample the co-localization ratios of the EVs signal with respect to CARS and SHG signals and vice versa were all extracted, in order to understand how the EVs signal overlap the CARS (cells) and the SHG (collagen, extracellular matrix) signals over the time. The co-localization ratio was computed evaluating the ratio of the total pixel in common between the two compounds and the total number of reference compound pixels (in this work the first compound nominated is the reference). These ratios were evaluated at each Z slice of the overall Z stack and the maximum and the average values were computed.

## 3. Results

### 3.1. ASC-EVs Showed Characteristic Size, Morphology and MSC-EVs Markers

**Figure 1:**
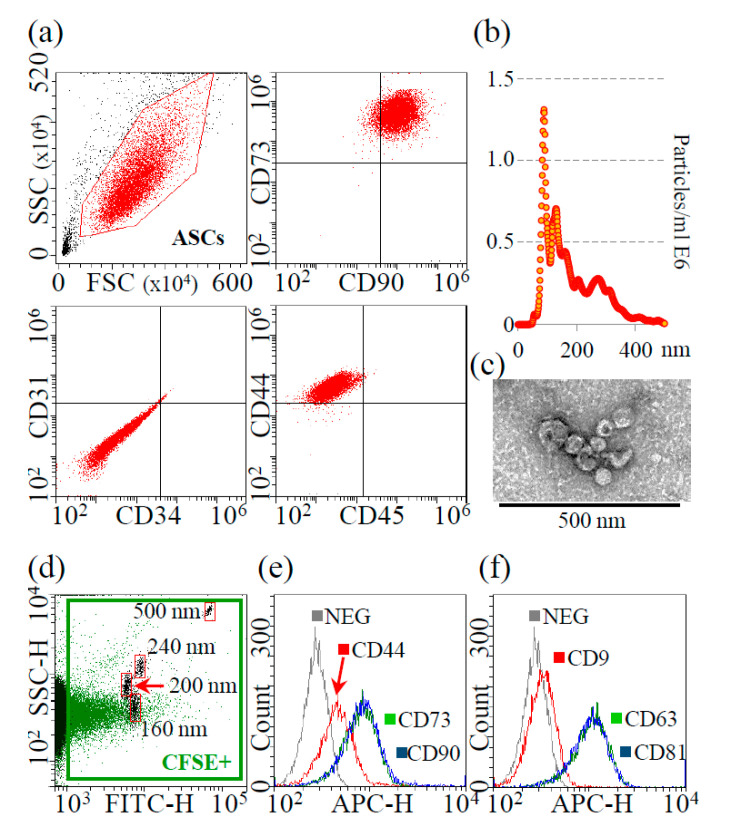
Characterization of ASCs and ASC-EVs. (a) Flow cytometry analysis of MSC (CD73, CD90 and CD44) and hemato-endothelial (CD31, CD34 and CD45) markers, presence and absence, respectively, confirming ASCs identity. Representative plots are shown. (b) Representative NTA plot of ASC-EVs; (c) Transmission electron micrographs of ASC-EVs showing particles with characteristic cup-shaped morphology. (**d**) Flow cytometry of FITC-labeled calibrating nanobeads (framed in red, from 160 to 500 nm) assuring calibration of flow cytometer and comparison with CFSE-labeled ASC-EVs (framed in green); (e) Presence of MSC-markers CD44, CD73 and CD90 on CFSE-labeled ASC-EVs. Representative plot is shown under the FITC + gate of EVs + CFSE; (f) Presence of EV-markers CD9 (weak), CD63 and CD81 on CFSE-labeled ASC-EVs. Representative plots are shown under the FITC + gate of EVs + CFSE.

MSC cell-surface antigens CD73, CD90 and CD44 were highly expressed in ASCs, whereas hemato-endothelial markers CD31, CD34 and CD45 were absent (Figure 1a).

Nanoparticle tracking analysis showed that isolated EVs were within the extracellular vesicle dimensional range (mode of 90.6 ± 2.0 nm), with >68% resulting below 200 nm and a D50 of 151.4 ± 3.0 nm (Figure 1b). Additionally, EV batches resulted to have 2.3 × 10^9^ ± 0.5 particles/μg protein (N = 3, mean ± SD), being depleted of major contaminants, as protein aggregates or lipoproteins, that may alter size distribution profiling [37]. TEM supported the presence of nanoparticles with the characteristic cup-shaped morphology (Figure 1c for magnified particles, Figure S3 for the wide-field image). Eventually, the presence of a signal in the FITC channel confirmed EVs integrity due to cytoplasm and CFSE labeling retention after centrifugation and, by comparison with FITC-nanobeads (160 nm, 200 nm, 240 nm and 500 nm), NTA size-range data was again validated (Figure 1d). MSC-EV defining markers CD44, CD73 and CD90 were present (Figure 1e), as well as generic EV-markers CD63 and CD81 (Figure 1f). CD9, another postulated EV-marker, resulted expressed at very low levels (Figure 1f), as previously described for ASC-EVs [38] and fetal MSCs [39].

### 3.2. Endpoint Incorporation Showed ASC-EVs Full Migration in Chondrocyte Micromass

**Figure 2:**
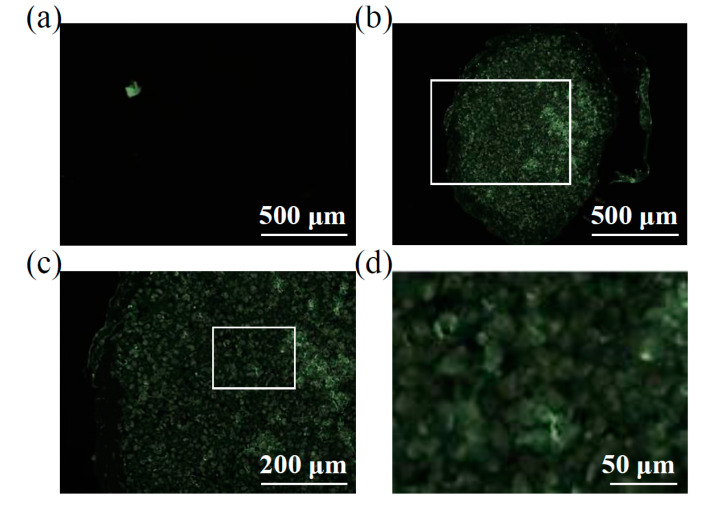
ASC-EVs endpoint incorporation in chondrocyte micromasses. (**a**) Transversal section of a representative chondrocyte micromass not treated with CFSE-EVs showing absence of background fluorescence. A representative picture is shown. (**b** through **d**) Increasing magnification of a representative chondrocyte micromass after incubation with CFSE-EVs, with the original region of panel c depicted in panel b by the white square as well as the original region of panel d being squared in panel c. It is possible to observe a homogenous signal all over the micromass section, including the center of the pellet (**b**) and, with a higher magnification (**c** and **d**), fluorescence results associated with both chondrocytes and intercellular matrix. Representative pictures are shown.

CFSE-labeled EVs were incubated with chondrocyte micromasses for 96 h and eventually fluorescence in the green channel scored in slices encompassing full-diameter pellets (Figure 2). With respect to samples cultured without vesicles (Figure 2a), ASC-EVs allowed for a homogenous and positive signal all over the micromass section, including the center of the pellet (Figure 2b). With a higher magnification (Figure 2c), it was possible to detect fluorescence associated with the chondrocytes and a faint inter-cellular and matrix related staining, with no signal intensity difference between the edge and the depth of the micromass. Overall, with standard techniques, in 96 h, ASC-EVs fully penetrated the micromass up to the central area, positively staining the chondrocytes. This suggests an active EVs migration that was not blocked by matrix structures, although with this technique it was not possible to follow neither the sharp kinetics nor the dynamics of live cells.

### 3.3. Time-Lapse Showed a Fast Penetration and Specific Pattern of ASC-EVs Incorporation in Chondrocyte Micromass and Cartilage Explants

**Figure 3:**
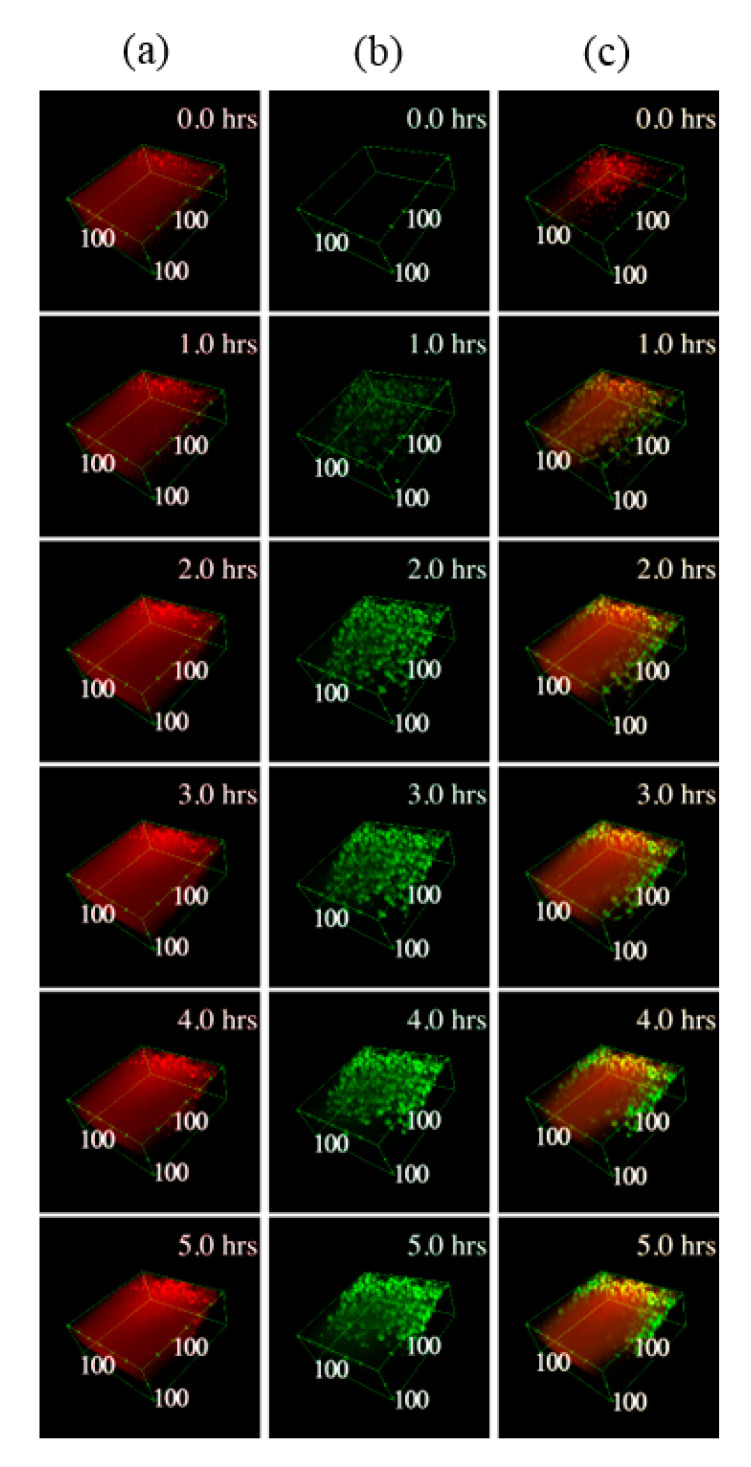
Three-dimensional (3D) reconstructions of the pellet micromass during the time-lapse. In column (**a**) the CARS signal at 2848 cm^−1^ originating mainly by the cells lipid structures is shown in red, in column (**b**) the TPEF signal related to the EVs fluorescence is shown in green, while in column (**c**) the two channels are merged. In the top right corner of each image is depicted the related timeframe and close to the boundary box are shown the dimensions in μm starting from the origin vertex.

**Figure 4:**
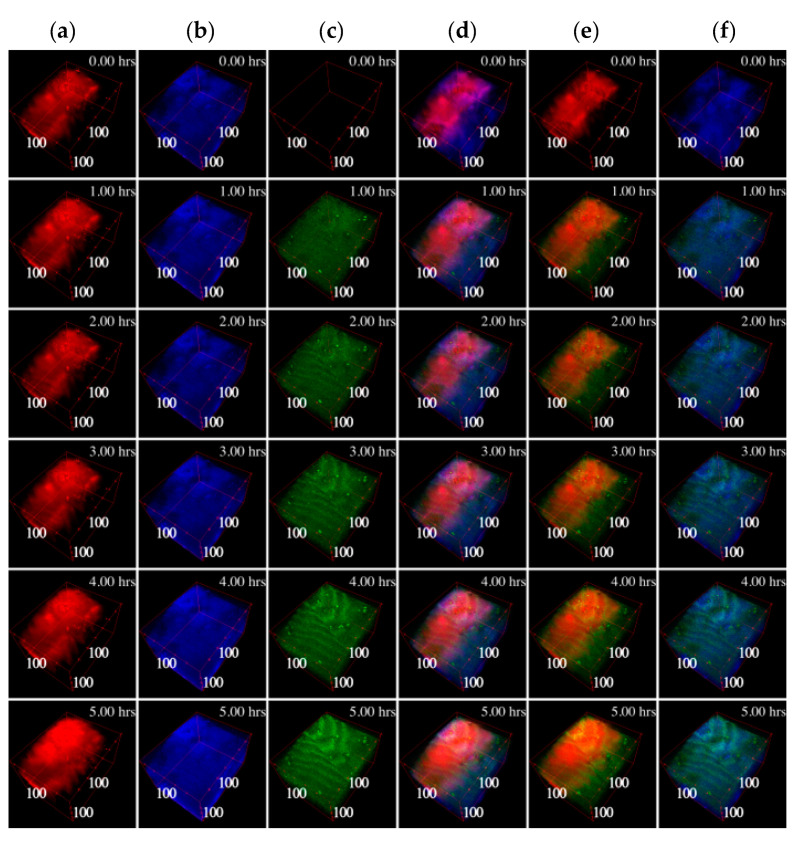
3D reconstructions of the cartilage during the time-lapse. In column (**a**) the CARS signal is shown in red at 2848 cm^−1^ originating mainly in the cells lipid structures; in column (**b**) the SHG signal generated by the collagen is shown in blue; in column (**c**) the TPEF signal related to the EVs fluorescence is shown in green. In column (**d**) all the three signals are visualized together, while in column (**e**) only the CARS and the TPEF are visualized, and in column (**f**) only the SHG and the TPEF are visualized. In the top right corner of each image the related timeframe is depicted and close to the boundary box the dimensions in μm are shown starting from the origin vertex.

In both cartilage explants and chondrocyte micromasses, EVs penetrated into the tissue, as clearly emerging from the 3D reconstructions of the samples (Figure 3 and Figure 4). In micromasses, EVs interacted with the cells quickly and homogenously, appearing more and more visible, defined and diffused within the matrix (Figure 3). The collagen signal was weak due to its paucity. When assessing the incorporation of EVs in the cartilage explants (Figure 4), it was possible to note that already after one hour, and along all the 5 h time-lapse, the EVs (green TPEF channel) co-localized within the collagen (blue SHG channel), as well as within the organic molecules in which the CH_2_ functional group is highly concentrated (mainly the cellular lipid structures imaged through the CARS red signal). Analyzing the samples at the different times of incubation, the average co-localization ratio of the EVs with respect to both collagen and lipid structures was quite constant (69.2 ± 1.8% and 90.4 ± 1.5%, respectively). Therefore, the vast majority of the EVs signal, as well as the particles entered the tissue, overlapped with both the extracellular matrix and chondrocytes.

**Figure 5:**
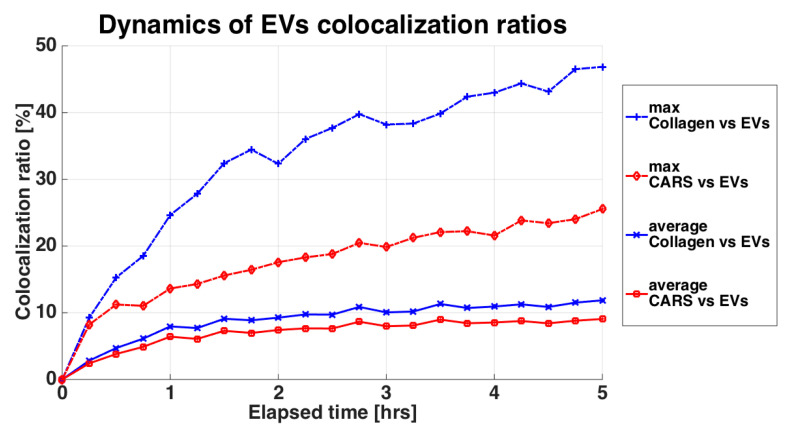
Variation of the co-localization ratios of the EVs with respect to the SHG signal and to the CARS signal. SHG is related to the collagen and thus the extracellular matrix, shown in blue. CARS is related to the lipid cell structures, shown in red. The curves indicate the maximum co-localization value and the average co-localization value computed from the set of Z slices composing the Z-stack for each timeframe.

Performing the co-localization analysis on the other way round, that is, taking as reference the collagen (SHG) or the lipidic structures (CARS) occupancies, both the maximum and the average co-localization ratios of the collagen versus the EVs were higher than those related to the cells (Figure 5). Focusing on the maximal values, the co-localization ratio of the collagen at the end of the 5 h-experiment nearly doubled the chondrocyte one, suggesting that EVs are absorbed by the extracellular matrix at a faster pace than their detected interaction with cellular component. Overall, cartilage explants and chondrocyte pellets behaved similarly, making the results obtained in the two models comparable. In the Supplementary Materials from the boxplots in Figure S4 it is possible to observe the co-localization statistical variability between the Z slices through the Z stack for each timeframe.

**Figure 6:**
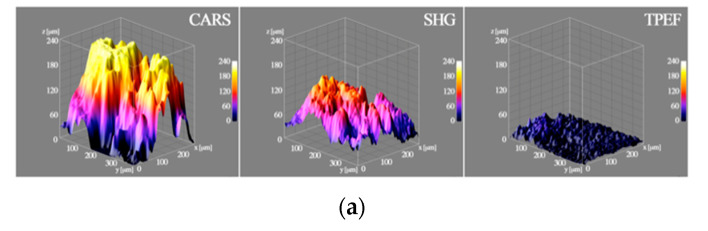

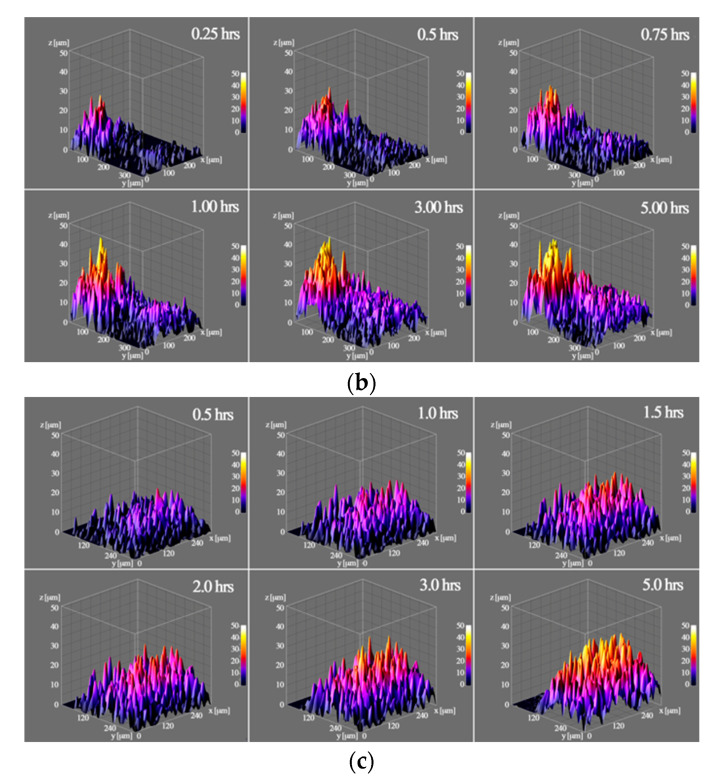
3D surfaces of the thicknesses extracted from the acquired and processed data. (**a**) The 3D surface on the left is related to the thickness of the cartilage cell structure computed from the CARS signal, in the center, the 3D surface is related to thickness of the collagen and thus the ECM from the SHG signal, while on the right the 3D surface is related to the EV penetration depths in the cartilage after 5 h from the TPEF signal; (**b**) the 3D surfaces show the dynamic of the EV penetration in the cartilage during the time-lapse; in the top right corner of each image the related timeframe is depicted; (**c**) the 3D surfaces show the dynamic of the EV penetration in the chondrocytes micromass during the time-lapse, in the top right corner of each image the related timeframe is depicted.

In Figure 6a, at the end of the 5 h time-lapse experiment, the 3D surfaces extracted from the cartilage showed the thickness referred to the CARS, SHG and to the TPEF. In the first 5 h, EVs started to penetrate the surface of the tissue (between 20 and 40 μm), without reaching the full thickness, measured with the CARS signal, which gave a more comprehensive idea of the entire volume. Dissecting the time kinetics, in Figure 6B and C it is possible to observe the EV penetration at different time frames in both cartilage and pellet, with the box of the 3D surfaces displaying the height of about 50 μm to facilitate comparison. In both models, the thickness of the portion of sample increased with time, confirming that the EVs were able to penetrate. In the cartilage explants (Figure 6b), EVs penetrated the tissue, with the inhomogeneity of the surface shape possibly being related to the sample morphology. Furthermore, in part of the cartilage, the EVs penetrated 25–30 μm already in the first hour of incubation, and just 15 min after the EVs were added to the culture medium, their presence and penetration were clearly visible (first image in the top left in Figure 6b). Similarly, in the chondrocyte pellet (Figure 6c), the EVs penetrated quickly, being clearly detectable already from the first time step, after just 30 min. The penetration appeared more homogenous through the micromass than in the cartilage model, probably because of the more homogenous and smoother surface created by the pellet generation technique. As in the cartilage explants, at the end of the 5 h time-lapse the EV depth reached 30–40 μm.

**Figure 7:**
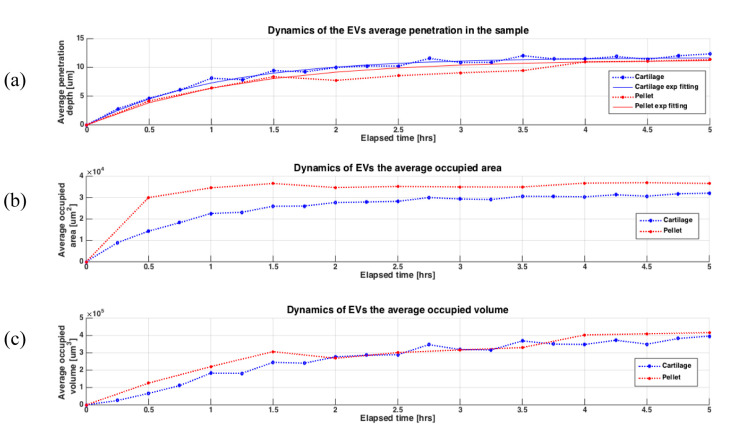
Dynamics of the EV uptake in the samples during the time-lapse experiment in terms of the average thickness of penetration, the average occupied area and the average occupied volume. (**a**) the dotted curves show the average EV penetration depth in the cartilage (blue) and the chondrocytes pellet (red), while the continue curves plot the related exponential fit for the cartilage (blue) and the chondrocytes pellet (red); (**b**) the dotted curves show the average occupied area of the EVs in the cartilage (blue) and the chondrocytes pellet (red); (**c**) the dotted curves show the average occupied volume of the EVs in the cartilage (blue) and the chondrocytes pellet (red).

The 3D surfaces also show the local inhomogeneity of the penetration depths due to the nature of the sample (i.e., the presence of cells or ECM). Figure 7 shows the dynamics of the EV uptake in terms of the average penetration depth, the occupied area and the occupied volume. The statistical distributions of the penetration depths at each timeframe for both the pellet and the cartilage are shown in Figure S5. The EV uptake exhibited in both experiments a similar behavior, with very similar results, making the two models comparable. The statistical variation also explains the roughness and the lack of homogeneity of the penetration depths. Furthermore, in the cartilage explants, EVs showed a slightly faster penetration than in the pellet, while the occupied volume had very similar values. Regarding the occupied area, the EVs spread sooner and larger in the pellet than in the whole cartilage. However, this could be explained by the more intense TPEF signal in the pellet leading to a higher sensitivity able to detect EVs in the pellet at a lower concentration than in the cartilage.

Eventually, the average penetration depth data were used to obtain the related exponential fitting curves of Equation Z_p_ (t) = Z_f_ (1 − e^−t/τ^), with two coefficients Z_f_ and τ that are, respectively, the final penetration depth and the uptake time-constant. The fitting output results gave similar coefficients for the cartilage explants and the micromass with Z_f_ of about 11.74 μm and 11.35 μm, respectively, and a transient time-constant τ equals to 1.03 h and 1.21 h, respectively. These results confirmed that in the cartilage the uptake was slightly faster than in the pellet, and that the EVs reached about 90% of the final transient average penetration depth in both models in slightly more than 2 h.

## 4. Discussion

The innovative imaging method used in this work allowed to observe, for the first time, both the gross and the finely-tuned related kinetics of ASC-EV penetration in chondrocyte micromasses and cartilage explants from osteoarthritic patients. These results confirm the ability of MSC-EVs to influence cartilage tissue/chondrocytes embedded in ECM, acting on the restoration of chondrocytes and overall cartilage homeostasis at all the tissue depths.

The release of different factors through EVs may orchestrate the main therapeutic mechanisms of action of MSCs [40]. Therefore, the use of MSC-EVs might provide considerable advantages over their counterpart living cells, by limiting undesirable side effects. In the orthopedic field, although a large number of registered MSC-based clinical trials (17%) is related to musculoskeletal conditions (https://clinicaltrials.gov/, accessed July 2020), no orthopedic study involving MSC-EVs is currently ongoing. Nevertheless, promising outcomes of in vitro and preclinical studies in OA models have been reported. In vitro, EVs obtained from bone marrow derived mesenchymal stromal cells (BMSCs) protected chondrocytes from apoptosis and increased the expression of type II collagen and aggrecan, while inhibiting catabolic (MMP-13, ADAMTS5) and inflammatory (iNOS) markers [6]. Similar, or even better, results were obtained by increasing the amount of EV-embedded miRNAs. Pre-conditioning of BMSCs with TGF-β up-regulated the EV incorporation of several miRNAs, including miR-135b, which promoted chondrocyte proliferation [41]. With a more refined strategy, such as the overexpression of a single miRNA by transfection, BMSC-EVs shuttling higher levels of miR-92a-3p increased the chondrogenesis [42], whereas synovial-MSC-EVs harboring up-regulated miR-140-levels stimulated the proliferation and migration of chondrocytes [8]. Oftentimes, these particles, both naturally released and artificially engineered, allowed for encouraging preclinical outcomes. In collagenase-induced OA model in mice, natural or miR-92a-3p MSC-EVs suppressed cartilage degradation [6,42]. In rat OA model, MSC-EVs overexpressing either miR-135b or miR-140 promoted cartilage tissue repair with very mild joint wear and cartilage matrix loss, without reducing aggrecan expression nor increasing type I collagen expression [8,41]. 

In the mentioned studies, the action of MSC-EVs was meant to be a direct interaction with chondrocytes, allowing the release of bioactive molecules such as miRNAs, able to modulate recipient cells by altering mRNAs and related pathways stability [43]. Despite this intriguing hypothesis, the proof of this mechanism relied on EVs incorporation in 2D cultured cells, which are, therefore, far from the 3D real environment of cartilage tissue. In this perspective, in 2D cultures, all the cells are directly exposed to EVs in the medium, whereas in a joint the cartilage cells embedded in the ECM and distributed within the different tissue layers would not be reached by EVs in the same ways. [44]. To improve the knowledge in term of EVs incorporation in 3D structures, we recently proposed a microfluidic device where OA chondrocytes were cultured in 3D conditions allowing a clear detection of their tridimensional morphology in a fibrin-based matrix, and compared this system with 2D cultures [45]. In this model, ASC-EVs injected in the 3D device quickly and homogeneously permeated the intercellular matrix, also far from the injection site, and they were internalized into the chondrocytes. Moreover, the presence of the fibrin-based matrix, absent in 2D cultures, suggested for this structure a role as an “EV sponge” able to both increase the local concentration and reduce the Brownian motion of particles around the cells, thus enhancing their uptake. However, the microfluidic model required to fix and stain samples at each time point of analysis, therefore reducing the continuity of a time-lapse analysis on living cells. Moreover, even if it resembles the cartilage matrix, the 3D microfluidic device based on the fibrin-gel technology cannot replicate the complexity of the native cartilage composed of several components, such as, among others, type II collagen and an interlocking mesh of fibrous proteins and proteoglycans, hyaluronic acid and chondroitin sulphate [44].

To overcome these limitations, in the herein presented study we analyzed the kinetics of ASC-EVs in micromass, the most commonly used in vitro model of 3D chondrocyte culture, and cartilage explants, even more closely resembling a real joint. Notably, the models gave rise to similar outcomes, suggesting that the presence of a “cartilage-like” structure is sufficient for reliable EV-based incorporation studies, for those laboratories that do not have easy access to patients’ tissues but mainly to cultivated cell lines, even if limiting factors of micromasses have to be taken into account. To visualize EV uptake, a sophisticated multimodal non-linear optics microscopy was finely tuned, based on well-established confocal laser scanning microscopy technology that was successfully used to interrogate cellular imaging and intracellular trafficking of fluorescent nanoparticles in 2D systems [46]. The herein proposed improved technical approach relied on the capacity to collect cellular lipidic structures (in chondrocytes membranes) with CARS, collagen and thus the extracellular matrix with SHG and FITC-like signals (CFSE-EVs) with TPEF techniques. The combination of CARS and SHG allowed analyzing micromasses and explants without the need of sample manipulation or staining before or during the co-culture. Moreover, a complete time-lapse analysis was possible, reducing the sample variability given by the analysis of different fixed and stained samples throughout the image collection process. Thus, single cells were observed, allowing for more precise results even in case of limited number of cells or samples. In these conditions, we confirmed a quick incorporation of EVs in the cartilage or micromass already observed in the microfluidic model. Already after few minutes, EVs were able to penetrate the samples for tens of μm. The dynamics of EVs co-localization ratios with cells or collagen suggested that EVs are quickly incorporated into the tissue surface, probably forming a concentration gradient, making EVs interaction with chondrocytes slower to reach both cell saturation and cells far from the tissue surface. As penetration is a mechanism that proceeds over time, although reducing its speed with the sample depth, the time-lapse data at 5 h corroborated the full staining of chondrocytes in the micromass observed at 96 h by conventional microscopy on fixed samples. Therefore, it is presumed that extracellular vesicles will be able to reach not only the surface of cartilage but also the intermediate and deepest areas to orchestrate healing mechanisms, although further experiments will be needed. 

A limitation of this work is the lack of bony structure in the 3D model. Due to the importance of the bone component for the homeostasis of the osteochondral unit, in the future it should be useful to also assess the EV penetration in bone, even if the little transparency of bone to the excitation wavelengths should be considered. From the point of view of the microscopy technique, the main limitations are related to the transmission of the excitation wavelengths and of the generated signal through the sample. Very thick samples could prevent the transmission of the optical signals, decreasing the measurement sensitivity and the image contrast. In principle, some of these issues could be solved by collecting the generated signal in the epi-direction. In living animal models experiments, it should also be considered to immobilize the animal to avoid blurring effects on the images.

This work is a proof of concept for the use of a novel and sophisticated microscopy technique on living cells, thus, future studies will be needed in conditions resembling the joint environment more closely. In particular, similar incorporation studies should be performed on synovial membrane samples as that plays a very important role in OA pathogenesis and progression. Furthermore, other cutting-edge and powerful technologies such as magnetic resonance or photoacoustic to highlight cell/tissue interaction with nanoparticles will be of crucial importance, as recently demonstrated in vivo to follow in tissue retention of pathogen-targeting phototheranostic nanoparticles to defeat methicillin-resistant *Staphylococcus aureus* [47].

## 5. Conclusions

This report showed a novel microscopy technique to monitor in real-time the diffusion kinetics of EVs in structures characterized by a complex extracellular matrix such as cartilage. Additionally, in both cartilage explants and chondrocyte micromasses, EVs were confirmed to quickly penetrate the surface, possibly reaching the deepest areas within few hours or, presumably, days depending on the tissue depth. The herein proposed technology may be used to monitor the EV penetration in all the tissues where a complex extracellular network is present, and to obtain data resembling physiological conditions. Eventually, these data support the hypothesis of the capacity of MSC-EVs to influence the chondrocytes embedded in their native ECM by active interaction and eventual therapeutic cargo release, supporting the feasibility of these advanced medicinal products as off-the-shelf options for the treatment of osteoarthritic joints and other diseases with similar characteristics. 

## Supplementary Materials

The following are available online at https://www.mdpi.com/1999-4923/12/8/734/s1, Figure S1: Simplified schematic of the microscope optical system. Figure S2: Example of an acquired Z slice image of the EVs during the time-lapse experiment. Figure S3: Wide-field of Figure 1c TEM image of ASC-EVs. Figure S4: Dynamic and statistical variation of the co-localization ratios of the EVs. Figure S5: Statistical distributions of the EV penetration depths.
