# Supplementary material for: In Vitro Study of Extracellular Vesicles Migration in Cartilage-Derived Osteoarthritis Samples Using Real-Time Quantitative Multimodal Nonlinear Optics Imaging
Source: Pharmaceutics. 2020 Aug 5;12(8):734. doi: 10.3390/pharmaceutics12080734 (PMC7464389; doi:10.3390/pharmaceutics12080734)
Supplement: Supplementary file 1 [file pharmaceutics-12-00734-s001.pdf]

pharmaceutics

MDPI

Supplementary Materials: In Vitro Study of Extracellular Vesicles Migration in Cartilage-Derived Osteoarthritis Samples Using Real-Time Quantitative Multimodal Nonlinear Optics Imaging

Leonardo Mortati, Laura de Girolamo, Carlotta Perucca Orfei, Marco Viganò, Marco Brayda-Bruno, Enrico Ragni and Alessandra Colombini

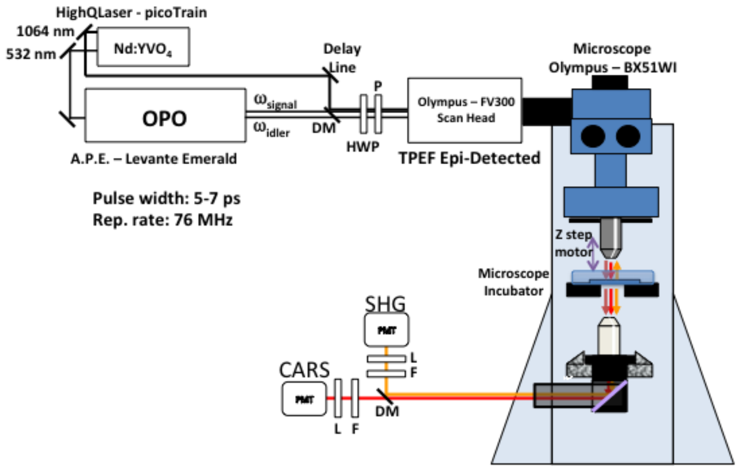

Figure S1. Simplified schematic of the microscope optical system. The abbreviations in the figure correspond to Dichroic Mirror (DM), Half Wave Plate (HWP), Polarizer (P), Lens (L), Filters (F), PhotoMultiplier Tube (PMT).

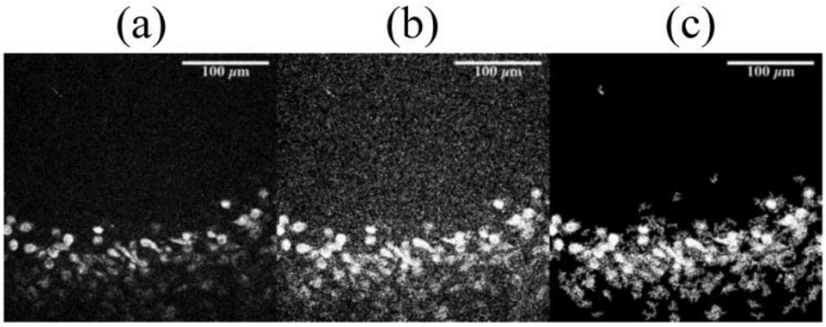

Figure S2. Correction of the background autofluorescence and of the salt and pepper effect during the time-lapse experiment. (**a**) Example of an acquired Z slice image of the extracellular vesicles (EVs) during the time-lapse experiment; (**b**) Previous image after the threshold processing to separate the foreground from the background; (**c**) Previous image after the particle size filter processing that cleaned all the objects with an area smaller than 100 pixels.

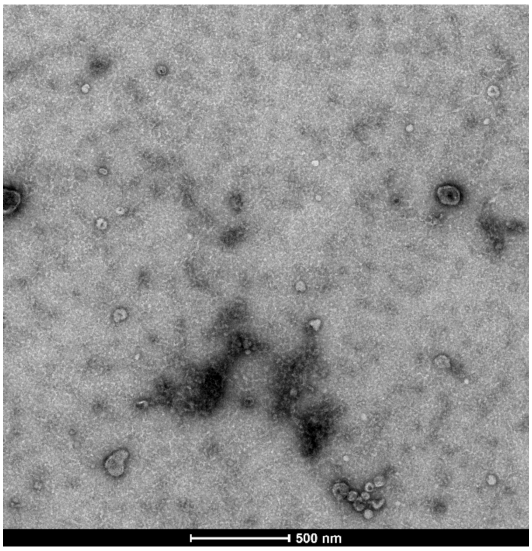

Figure S3. Wide-field of Figure 1c TEM image of ASC-EVs.

(a)

(b)

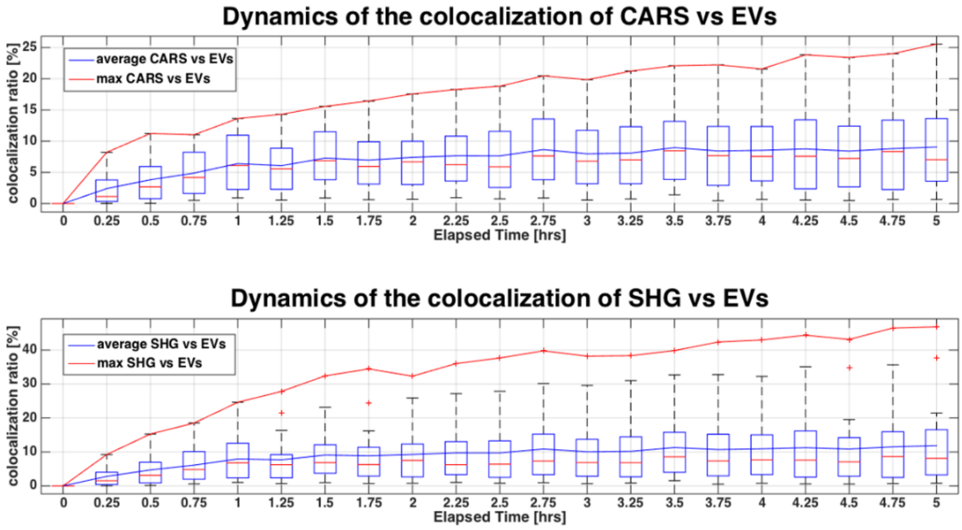

Figure S4. Dynamic and the statistical variation of the co-localization ratios of the EVs with respect to the CARS signal related to the lipid cells structures (**a**) and with respect to the SHG signal related to the collagen and thus the extracellular matrix (**b**). Each plot shows the statistical boxplot related to the data population of that specific timeframe, together with maximum and average values. On each box, the central mark is the median, the edges of the box are the 25th and 75th percentiles, the whiskers extend to the most extreme data points not considered outliers and outliers are plotted individually.

(a)

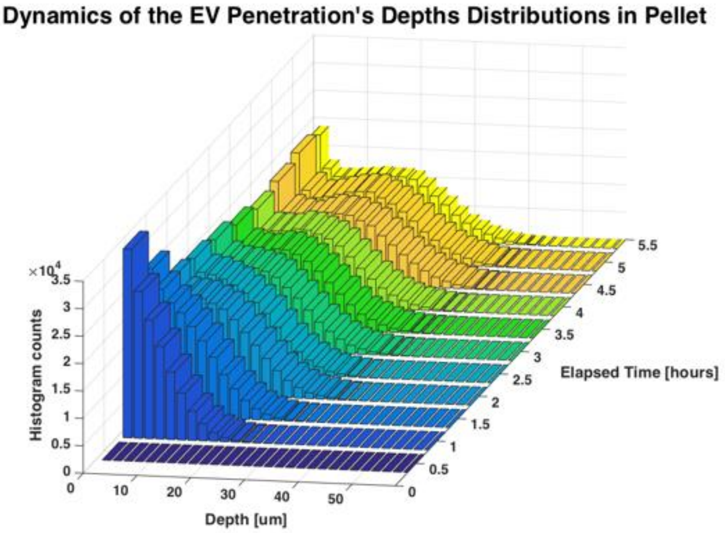

(b)

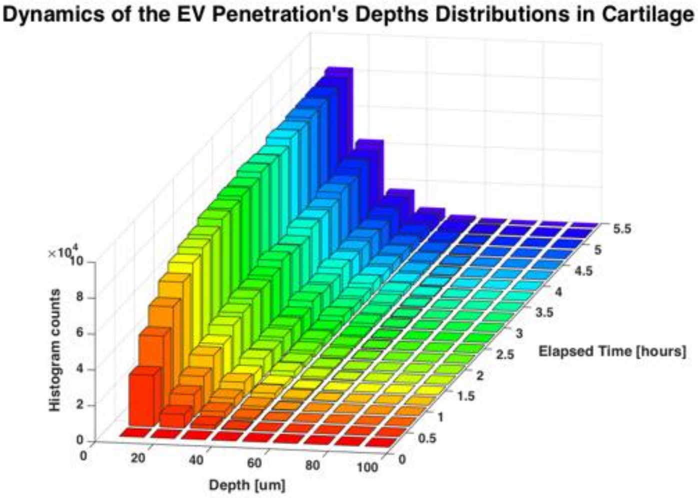

Figure S5. Statistical distributions of the penetration depths for each timeframe during the time-lapse experiment. In the 3D histograms of (**a**) pellet and (**b**) cartilage, the counts represent the number of pixels related to each penetration depth and their sum multiplied to the XY pixel area leads to the total EVs occupied area. In the same way the sum of the number of voxels, also taking into account the relative depths, leads to the total EVs occupied volume. The shapes of the distributions are not strictly Gaussian in both cases and for the pellet it could be perceived a bimodal characteristic.
